# The Knowledge, Attitude, and Perception towards Epilepsy amongst Medical Students in Uyo, Southern Nigeria

**DOI:** 10.1155/2015/876135

**Published:** 2015-03-31

**Authors:** Bertha C. Ekeh, Udeme E. Ekrikpo

**Affiliations:** Department of Internal Medicine, University of Uyo, PMB 1017, Uyo, Nigeria

## Abstract

*Background and Aim.* Epilepsy remains a stigmatized disease especially in Sub-Saharan Africa. Lack of information and illiteracy has been blamed as the cause of the stigmatization. This stigmatization stems from the fact that the traditional African belief views epilepsy as a spiritual disease. We studied the knowledge, attitude, and perception towards epilepsy amongst medical students comparing the knowledge of the clinical students with that of the basic medical (preclinical) students. *Methodology.* The participants were medical students in University of Uyo. We administered questionnaires which explored the knowledge of etiology (perceived and medically proven). We studied the beliefs in infectivity of epilepsy, treatment together with their attitudes, and perception to persons with epilepsy. *Results.* Most of the participants do not have a good knowledge of epilepsy. The knowledge, however, was much better amongst the clinical students. There is some difference in the attitudes of the clinical students compared with the basic students. *Conclusion.* There is a knowledge gap in epilepsy even amongst medical students. Participants still harbor the traditional African beliefs that epilepsy is a spiritual disease. Mercifully, the knowledge is better amongst the clinical students. This is not surprising since the clinical students have had clinical exposure to epilepsy.

## 1. Introduction

Epilepsy is the most common noninfectious neurologic disease in developing African countries which include Nigeria [1]. At a conservative estimate, 50 million people worldwide suffer from epilepsy. It has been shown that as much as 80% of persons with epilepsy live in the developing world [2]. There is an annual incidence of 20–70 cases per 100,000 [3] with a point prevalence of 0.4–0.8% [4]. The reported prevalence of active epilepsy in the developing countries is between 5 and 10% per 100 persons [5]. It, however, varies in the general population being the highest in children, plateaus between the ages of 15 and 65 years, and rises again in the elderly [3–5]. Even with this high prevalence rates, there is yet likelihood that the reported rate is the “tip of the iceberg” as chances of underreporting are high [4, 5].

Historically, epilepsy was believed to be a sacred disease that is the result of the invasion of the body by a god. It was thought that only a god could deprive a healthy man of his senses, throw him to the ground, convulse him, and then rapidly restore him to his former self again [6]. Regrettably, this historical legacy has continued to influence public attitude to epilepsy making it a dreaded disease. These beliefs have resulted in patients with epilepsy (PWE) being ostracized, stigmatized, and misunderstood. The social implications are serious. For instance, in Madagascar, patients with epilepsy are refused burial in the family grave [7]. In many African countries, the PWE is an outcast as Africans believe epilepsy is a spiritual disease. According to the African belief, other possible etiologies include witchcraft and poisoning. A combination of these traditional beliefs, poverty, lack of medical care, and inability to fulfill their social roles has a negative impact on the lives of people living with epilepsy (PWE) [6].

One aspect of the sociocultural belief is that many people in Africa believe epilepsy to be contagious and that it can be spread by urine, saliva, flatus, or faeces excreted at all times or during a convulsion [8–10]. This results in isolation and unwillingness of witnesses to touch and protect the patient from injury during a seizure. Epilepsy is also believed to be transferable from one person to another by various routes. This leads to courtesy stigma where relatives, friends, and companions of persons with epilepsy are stigmatized as well. In view of the foregoing, most patients suffering from epilepsy in African countries prefer anonymity because of the stigma attached to the disease [3].

The sociocultural belief also greatly influences the health seeking behaviour and the management thereafter. It is estimated that 80% of the population of a developing country like Nigeria live in the rural areas and lack access to western type hospitals but rather seek help from traditional healers, churches, and others [11]. Anecdotal reports show that some do not believe that epilepsy can be treated medically.

In 1997, the three leading organizations in the field of worldwide epilepsy started working together to reach better general awareness, the ILAE/IBE/WHO Global Campaign against Epilepsy. Their mission statement is* to improve the acceptability, treatment, services, and prevention of epilepsy worldwide*. Activities include the organization of regional conferences with the development of Regional Declarations which are used in communication with stakeholders, regional reports, and demonstration projects [12]. In the African Declaration governments are called to develop national plans for the education of health workers, patients, and the general public; to eliminate discrimination; to promote interactions with traditional health systems; and to declare a National Epilepsy Day [13].

The study was carried out in University of Uyo in Akwa Ibom state, Nigeria. The experience of the authors in the practice has shown that there is a very poor knowledge of epilepsy in the state with much stigmatization. Surprisingly, the poor knowledge is seen in all socioeconomic classes. It is not unusual to see educated persons who stigmatize persons with epilepsy. One of the authors' patients had to stop working after she had a seizure in the work place because she could not bear the stigma, shame, and humiliation thereafter. The authors have patients with epilepsy who were withdrawn from school by their educated parents.

Medical students make up the upper echelons of the undergraduate students and the society in general. Most of their knowledge and perception of diseases before exposure to medical education originate from the beliefs of the society in general. We undertook this study to examine the perception of epilepsy among medical students. We compared the perception of the students from basic medical sciences (preclinical) who still have a preconceived notion of epilepsy with that of the clinical students whose preconceived notions have been altered to some extent by clinical exposure. All the participants are not in the same grade. The basic medical students are preclinical students who have had no clinical exposure whatsoever. Their knowledge of epilepsy, therefore, represents that of the general educated part of the community (like university undergraduates). The clinical students, however, have commenced clinical training. They have had lectures on epilepsy and seen the management of some of the persons with epilepsy. Consequently, these clinical students are the only participants who have been exposed to the correct knowledge of epilepsy. All the participants are also of similar intellect. Most of them are from similar background and at such have the same beliefs and values. Clinical exposure is the only difference between the two groups. The knowledge gained from this study will be used in the planning public enlightenment campaigns. The campaigns will start from not just medical students but also other undergraduate students. Having being educated about epilepsy, they will be empowered to teach others in their families, communities, and society as a whole.

## 2. Methodology

This was a cross-sectional survey carried out in the University of Uyo, Akwa Ibom state. Akwa Ibom is a state in Nigeria named after the Qua Iboe River. It is located in the coastal Southern Nigeria part of the country, lying between latitudes of 4°321 and 5°331 north and longitudes of 7°251 and 8°251 east. The state is bordered on the east by Cross River State, on the west by Rivers State and Abia State, and on the south by the Atlantic Ocean and the southernmost tip of Cross River State. The major cities in the state are Uyo, Ikot Ekpene, Eket, and Oron. Uyo is the capital city.

University of Uyo medical school though relatively new has graduated four sets of medical doctors. The study population was made up of medical students in the university. Both clinical and basic medical students were studied. Questionnaires were distributed to the participants while they were in the classrooms. All the medical students who were met in their classroom were studied. The questionnaires were distributed to all the participants. All the participants who gave their consent to willingly take part were included in the study. All the students who declined to participate in the study were excluded.

### 2.1. Analysis of Result

Categorical data was presented as frequencies and percentages and Pearson's chi-square (*χ* ^2^) eight used to compare proportions for the two groups of respondents. A *P* value < 0.05 was deemed statistically significant. The whole analysis was performed using STATA 10, StataCorp, Texas, USA.

## 3. Results

A total of 250 questionnaires were distributed. Two hundred and thirty-two of these were retrieved. The remaining eighteen were not returned. The response rate was 92.8%. There were a total of 121 basic medical students and 111 were clinical students. The age range was 16–43 years. The age group (20–29) was the mean, median, and also mode. There were 144 (62.61%) males and 86 (37.39%) females. Two participants failed to write their sex. Eight (3.48%) of the participants were married while the rest were single. There were two hundred and thirty (99.18%) Christians while the remaining two participants (0.82%) were Muslims.

One hundred and seventy-eight (76.72%) participants were from Akwa Ibom state while 41 (17.67%) others were from the neighbouring states. The remaining 13 (5.6%) participants were from more distant states. As much as 201 (87.02%) of the participants were of Ibibio, Annang, or Ibo ethnicity with 119 (51.52%) of Ibibio ethnicity alone.

### 3.1. Knowledge of Epilepsy

Participants were asked whether they had heard of epilepsy. There were only two participants who had not heard of epilepsy (one from the basic participants and the other from the clinical participants). The rest of the participants had heard of epilepsy. A total of 108 (46.75%) of the participants know someone with epilepsy: 68 of these were clinical students (62.96%) as against 42 from the basic medical sciences (33.06%).

On the etiology of epilepsy, 18 (14.88%) of the basic students and 6 (5.41%) of the clinical students were positive that epilepsy was caused by evil spirits. Eleven (9.09%) of the basic students compared to 6 (5.41%) of the clinical students believe that epilepsy was caused by witches. Ten of the basic students as against only 3 (2.70%) of the clinical students identified palm oil as a cause.

The knowledge of the participants on the medical causes of epilepsy was also tested. In relation to trauma as the cause of epilepsy, only 54 (44.63%) of the preclinical students were in the affirmative as against a whopping 92 (82.88%) of the clinical students.

Another large number of clinical students 95 (85.59%) know that birth injuries can cause epilepsy as against 54 (47.11%) of the basic students. Only 44 (36.36%) of the preclinical students were aware that infections can cause epilepsy as against 98 (88.29%) of the clinical students. On brain tumours, 98 (88.29%) of the clinical students were aware that brain tumours can cause epilepsy in comparison with 87 (71.90%) of the basic students. Concerning stroke, 63 (56.76%) knew that stroke can cause epilepsy in comparison with 52 (42.98%) of the basic students. The above information is represented in Table 1.

We also asked about the popular belief that epilepsy is transferable. Thirty-two (26.44%) of the basic students and 31 (27.92%) of the clinical students affirmed that epilepsy is transferable. There were twenty-seven (22.31%) of the basic students compared to 11 (9.91%) of the clinical students who felt that epilepsy was contagious, *P* = 0.002. Thirty (24.79%) of the basic students identified saliva as a route of transmission and 11 (9.91%) of the clinical students felt the same, *P* = 0.004. Forty-six (38.02%) of the basic students identified blood as a route as against 6 (5.41%) of the clinical students, *P* = 0.000.

Only 10 (8.26%) of the basic students identified urine as a route of transmission similar to 6 (5.41%) of the clinical students. In like manner, 6 (4.96%) of the basic students and 4 (3.60%) of the clinical students acknowledged faeces as a route of transmission.

On the possibility of epilepsy treatment, 88 (72.73%) were positive that epilepsy can be treated compared to 96 (86.49%) of the clinical students. Similarly, 83 (68.60%) of the basic students know that epilepsy can be treated in the hospital in comparison with 97 (87.39%) of the clinical students, *P* = 0.003.

The participants were asked whether they knew any antiepileptic drugs. A total of 68 (61.26%) clinical students knew at least one antiepileptic drug as against only 2 (1.6%) basic students, *P* = 0.000.

The most prevalent antiepileptic drug was carbamazepine named by 43 participants. Sodium valproate was the next prevalent drug mentioned by 27 participants followed by phenytoin by 22 participants. Other less known drugs were benzodiazepines mentioned by 9 participants, barbiturate which was named 8 times, and ethosuximide which was named 4 times. Newer antiepileptic drugs were not well known: levetiracetam thrice, lamotrigine twice, topiramate once, and vigabatrin once. Two of the basic medical students mentioned traditional herbs as drugs for epilepsy.

The response of the participants to a person having a seizure is captured in Table 2. In the same vein, the participants' relationship with a person with epilepsy is as captured in Table 3.

Furthermore, we asked the participants where they will take a relative with epilepsy for care.

Ninety-eight (80.99%) of the basic students and 110 (99.10%) of the clinical students will take such a person to the hospital, *P* = 0.000. As much as 58 (47.93%) of the basic students and 31 (27.93%) of the clinical students will take him to the church, *P* = 0.002. Nine (7.44%) of the basic students and 5 (4.45%) will take him to the traditional healer. Only 2 (1.65%) out of the basic students and 3 (2.70%) of the clinical students will hide him. Twenty (16.53%) basic students as against 4 (3.60%) of the clinical students will administer herbs, *P* = 0.002.

Concerning whether a PWE can have children, 105 (86.78%) of the basic students and 109 (98.20%) of the clinical students affirmed that a female PWE can have children, *P* = 0.004.

Similarly, 104 (85.95%) basic students and 109 (98.20%) of the clinical students were positive that a male PWE can have children, *P* = 0.002.

## 4. Discussion

The study showed the knowledge of the preclinical participants as against those in the clinical sciences. The knowledge of the participants in the basic medical sciences (preclinical) represents the knowledge of most undergraduates and as such the background knowledge in the educated in the society. The participants are of comparable ages. Most of the participants (76.72%) are from Akwa Ibom state. Akwa Ibom state has three major ethnic groups which are Ibibio, Annang, and Oron. The language, beliefs, and culture are basically the same, with subtle differences. These students coming from the same cultures and sociocultural beliefs, therefore, represent those of the entire ethnic groups in Akwa Ibom state, Nigeria. Some others (17.67%) are indigenes of nearby states mostly Cross River, Imo, and Abia and Rivers states. Only 13 (5.61%) participants were from distant states. The most predominant ethnic group was Ibibio with Annang second and Ibo third. We noted that the participants are mostly from the same states and ethnic groups, speak similar languages, and have lived in the same environment and such have the same cultural beliefs and values. They are also of similar intellect. Clinical exposure is the only difference between the two groups. We believe that the knowledge of these students may, therefore, represent the beliefs in these ethnic groups.

We found that all but two of the participants had heard of epilepsy. However, more of the participants who were clinical students knew at least one person with epilepsy. There were no available studies in university students in Nigeria or Africa for comparison. Most knowledge, attitude, and practice (KAP) studies have been on teachers and the community. We, however, found two KAP studies of epilepsy on university students in Jordan [14] and Malaysia [15]. In the study from Jordan, three major universities were studied and 500 questionnaires collected from each university. They showed that 77.6% had either heard or read about epilepsy [14]. The Malaysian study had 289 respondents and as much as 86.5% [15] had heard of epilepsy. These figures are in contrast with the findings in our study where all but two participants had heard of epilepsy. This may possibly be because epilepsy is commoner in a developing country like Nigeria, the country of this study [1, 2]. More so, the participants in those two studies were students from different courses whereas all the participants in our study were only medical students. In fact one of the studies concluded that participants from humanities had more negative attitudes towards PWEs [14].

We showed that a total of 108 (46.75%) of the participants know someone with epilepsy: 68 of these were clinical students (62.96%) as against 42 from the basic medical sciences (33.06%). This may mean that because of the lecture on epilepsy and also seeing patients with epilepsy the clinical students are more apt to recognize persons with epilepsy. We think that some of those clinical students probably only recognized their friend or relatives diagnosis after the clinical exposure because they now have a higher index of suspicion.

The traditional African belief is that epilepsy is a spiritual disease caused by evil spirits, witchcraft, and even excessive palm oil [3]. Interestingly, these participants which are medical students confirmed this much trumpeted anecdotal report. This belief was so irrespective of the clinical exposure the clinical students have had. Nevertheless, fewer of the clinical students held these sociocultural beliefs. It is noteworthy that about 20% of these participants were not sure whether these sociocultural beliefs were causes of epilepsy or not. The response of the clinical students compares favourably with the Malaysian students where only 5.3% thought epilepsy was caused by evil spirits [15]. The study from Jordan showed that a significant proportion of students thought that epilepsy could be caused by the evil spirit (31.5%) and the evil eye (28.1%) or that it could be a punishment from God (25.9%) [14].

The knowledge of known medical causes of epilepsy showed that the clinical students were much more knowledgeable in comparison with the basic medical students. Interestingly, there was no statistical difference concerning stroke as a possible cause of epilepsy but they identified head trauma, infections, birth injuries, and brain tumours as possible causes/precipitants. This is not surprising considering the fact that some of these clinical students had already had lectures or bedside teachings on seizures.

Epilepsy is being painted as a highly contagious disease. It is said to spread through saliva, urine, blood, and even faeces [7–9]. This is the reason persons with epilepsy may not get help while having a seizure. The ictal period is supposedly the stage when such a person is most infectious. We showed that more than a quarter of the participants (27.92% of the clinical and 26.45% of the basic students) believe epilepsy to be transferable from one person to another. There was no statistical difference between the clinical and basic medical students surprisingly. However, a higher percentage (22.31%) of the basic medical as against that (9.91%) of the clinical students felt that it was a contagious disease. We showed that the basic medical students who believe that epilepsy is contagious consider saliva and blood as the main routes of transmission buttressing the previously recorded beliefs [7–9].

The term transferable or transmissible means that the disease can pass from one person to another. Routes of transmission include vertical (mother to baby), sexual, blood, air borne, water borne, and others. A contagious disease, however, is a subset of a transmissible disease. This means that the disease can be spread by direct contact or contact with body fluids. The belief of a quarter of the participants that epilepsy is transferable or transmissible means that they think it can be transmitted sexually, vertically, and even through blood or inherited. This leads to the courtesy stigma seen in epilepsy. On being contagious, however, fewer of the clinical students believed that it can be contacted by direct contact (saliva, urine, blood, or faeces).

Our study showed that most of the participants consider epilepsy a treatable disease even though there was a statistical difference between the two groups with the clinical students more inclined to the belief that epilepsy is a treatable disease. We also found that most of the participants believe that epilepsy can be treated in the hospital with a statistical significance between clinical and basic medical students groups.

Even though both groups strongly believe that epilepsy can be treated in the hospital, most of the preclinical participants do not know any antiepileptic drugs. Amazingly some of the participants wrote traditional herbs as medications for epilepsy. Years of education have not wiped out this strong certainty that epilepsy can only be treated traditionally. An earlier Nigerian study reported a 47.6% usage of African traditional medicine and 24.1% combined usage of traditional and spiritual methods [16]. The clinical students showed much better knowledge. The commonest antiepileptic drug known to the participants is carbamazepine. This is closely followed by valproate and phenytoin. This is not surprising as these are the commonest medications used in our practice. Carbamazepine has gained wide acceptance as an efficacious antiepileptic drug [5]. Benzodiazepines and barbiturates are also mentioned but should be known by more participants since they are used to abort seizures in emergency situations.

We showed the response of the participants to a person having a seizure. Interestingly, 7.44% of the basic students will run away instead of helping the person. This buttresses the finding that a good number of the participants still consider epilepsy a contagious disease. Only one clinical student will run away. We found that more than a quarter of the participants in each group, 36.36% in the basic and 25.23% in the clinical students, will put an object in the mouth of the person. This ill-advised practice is very common in rural Africa. It usually causes a lot of injuries to the tongue, mouth, lips, and gullet. It is surprising that the participants who are educated undergraduates with some degree of enlightenment will carry out this barbaric practice.

Concerning the correct measures to take when someone is having a seizure, the clinical students were better. They were more likely to help a person having a seizure by turning him to the side, protecting him from injury and loosening anything tight around his neck. These actions access the willingness or reservations in touching persons who are having seizures. Surprisingly more of the basic students were more likely to assist the person to stand up after a seizure unlike the clinical students. More of the clinical students will reassure the person after a seizure.

On examining relationship with PWEs, we showed that more than 70% of the participants will not marry a PWE. The authors have been asked by intended spouses of PWEs whether epilepsy is sexually transmitted. We supposed that this fear of transmission could be the concern. However, it is impressive that a quarter of the clinical participants will marry a PWE. Being friends with PWEs seemed more tolerable though the clinical students were still more likely to be friends with PWEs. Even eating with PWEs is also considered tolerable by the participants. The participants are also very likely to engage in other activities like living, trading, working, and employing a PWE. A Nigerian study showed that majority of respondents harboured positive attitudes such as tolerance, kindness, and sympathy towards epileptics. Literate respondents were more likely to exhibit positive feelings towards epileptics when compared to illiterate subjects [17]. Could this mean that the ostracization is gradually reducing at least among the educated individuals? More studies will explore this possibility. The most common negative attitudes toward PWE in the study from Jordan were that the students would refuse to marry someone with epilepsy (50.5%) and that children with epilepsy must join schools for persons with disabilities (44.4%) [14]. More so, the participants from the humanities had more negative attitudes [14]. This highlights the impact of poor knowledge.

Once again, the response of the participants concerning their care of a PWE is interesting. It is surprising to note that even though a majority of the participants will take such a person to the hospital, not all of them will. Almost 20% of the basic students despite their university education will not take the person to the hospital. The high percentage of the participants who will take the person to church (47.93% for basic students and 27.93% for clinical students) is worthy of note. Epilepsy is majorly believed to be a spiritual disease. This background belief still colours the health seeking behaviour in these participants despite their level of education. Mercifully, only a negligible number will either take him to the traditional healer or hide him. Contrariwise, some of the participants will administer herbs to such a person. Generally, Africans have been shown to seek health care from alternate health practitioners [16, 18]. This, however, is surprising in this study since the participants are medical students. In a community study in northern Nigeria, majority of respondents (47.0%) opted for spiritual healing. This was followed by orthodox medical care (34.0%) and the use of traditional herbal medicines (19.0%) [17]. The use of spiritual healing is even worse in the study from Jordan where epilepsy's most commonly reported treatment methods were the Holy Quran (71.4%), medications (71.3%), and herbs (29.3%) [14].

Finally, we found that most of the participants are aware that a PWE either male or female is capable of reproduction. This fear is one of the main reasons people do not want to marry a PWE. The second is that of the fear of possible heritability of this dreaded disease. The major limitation of this study is the number of participants. The next KAP study on epilepsy will involve other university undergraduates and the number of participants will be much larger.

## 5. Conclusion

Knowledge gap in epilepsy exists even amongst medical students. The knowledge of etiology is much poorer than expected. Granted, the clinical students show much better knowledge, but it seems that the preconceived sociocultural beliefs are still deeply rooted irrespective to the exposure to education and medical knowledge. The impact of sociocultural beliefs is still greatly seen in the perception of epilepsy even amongst the educated. Nevertheless, the attitude towards PWEs seems to be improving though still superior among the clinical students. The enormous work involved in awareness, education, and public enlightenment cannot be overemphasized.

## Figures and Tables

**Table 1:** Awareness and proposed causes of epilepsy.

	Heard	Know	Evil spirit	Witchcraft	Palm oil	Trauma	Birth injuries	Infections	Brain tumours	Stroke
Basic	120 (99.17%)	40 (33.05%)	18 (14.88%)	11 (9.09%)	10 (8.26%)	54 (44.63%)	57 (47.11%)	44 (36.35%)	87 (71.90%)	52 (42.98%)

Clinical	109 (98.2%)	68 (61.26%)	6 (5.41%)	6 (5.41%)	3 (2.70%)	92 (82.88%)	95 (85.59%)	89 (80.18%)	98 (88.29%)	63 (56.76%)

*P* value	0.946	0.000	0.007	0.040	0.003	0.000	0.000	0.000	0.002	0.109

**Table 2:** Reaction to a person having seizures.

	Run away	Put something in his mouth	Turn him to the side	Protect him from injury	Loosen anything on his neck	Help him stand after the seizure	Reassure him after the seizure
Basic	9 (7.44%)	44 (36.36%)	29 (23.97%)	70 (57.85%)	67 (55.37%)	61 (50.41%)	53 (43.89%)

Clinical	1 (0.9%)	28 (25.23%)	49 (44.14%)	100 (90.09%)	85 (76.58%)	38 (34.23%)	64 (57.66%)

*P* value	0.018	0.063	0.003	0.000	0.003	0.013	0.066

**Table 3:** Relationship with a PWE.

	Will you marry a PWE?	Will you be friends with a PWE?	Will you eat with a PWE?	Will you trade with a PWE?	Will you live with a PWE?	Will you work with a PWE?	Will you employ a PWE?	Are you afraid of a PWE?	Should a PWE go to school?	Is the PWE normal?
Basic	13 (10.74%)	93 (76.86%)	80 (66.12%)	98 (80.99%)	90 (74.38%)	97 (80.17%)	66 (54.55%)	26 (21.85%)	108 (89.26%)	57 (40.71%)

Clinical	29 (26.13%)	104 (93.69%)	97 (87.39%)	106 (95.50%)	92 (82.88%)	93 (83.78%)	70 (63.06%)	22 (19.82%)	107 (96.40%)	83 (59.29%)

*P* value	0.005	0.001	0.001	0.003	0.156	0.125	0.105	0.048	0.160	0.000

## References

[1] WHO (2004). *Epilepsy in the WHO African Region, Bridging the Gap: The Global Campaign against Epilepsy “Out of the Shadows”*.

[2] Baskind R., Birbeck G. L. (2005). Epilepsy-associated stigma in sub-Saharan Africa: the social landscape of a disease. *Epilepsy and Behavior*.

[3] Shorvon S. D. (1990). Epidemiology, classification, natural history, and genetics of epilepsy. *The Lancet*.

[4] Hauser W. A., Annegers J. F., Kurland L. T. (1991). Prevalence of epilepsy in rochester, minnesota: 1940–1980. *Epilepsia*.

[5] Olubunmi A. O. (2006). Epilepsy in Nigeria—a review of etiology, epidemiology and management. *Journal of Postgraduate Medicine*.

[6] Reynolds E. H., Trimble M. R., Reynolds E. W. (1988). Historical aspects. *Epilepsy, Behaviour and Cognitive Function*.

[7] Osuntokun B. O. (1978). Epilepsy in Africa. *Tropical and Geographical Medicine*.

[8] Sanya E. O., Salami T. A. T., Goodman O. O., Buhari O. I. N., Araoye M. O. (2005). Perception and attitude to epilepsy among teachers in primary, secondary and tertiary educational institutions in middle belt Nigeria. *Tropical Doctor*.

[9] Tekle-Haimanot R., Abebe M., Forsgren L. (1991). Attitudes of rural people in central Ethiopia toward epilepsy. *Social Science & Medicine*.

[10] Rwiza H. T., Matuja W. B. P., Kilonzo G. P. (1993). Knowledge, attitude, and practice toward epilepsy among rural Tanzanian residents. *Epilepsia*.

[11] Ogunniyi A., Osuntokun B. O. Epidemiology of neurologic illnesses in Africa.

[12] Birbeck G. L., Kalichi E. M. N. (2004). Epilepsy prevalence in rural Zambia: a door-to-door survey. *Tropical Medicine and International Health*.

[13] Atadzhanov M., Haworth A., Chomba E. N., Mbewe E. K., Birbeck G. L. (2010). Epilepsy-associated stigma in Zambia: what factors predict greater felt stigma in a highly stigmatized population?. *Epilepsy & Behavior*.

[14] Hijazeen J. K., Abu-Helalah M. A., Alshraideh H. A. (2014). Knowledge, attitudes, and beliefs about epilepsy and their predictors among university students in Jordan. *Epilepsy & Behavior*.

[15] Ab Rahman A. F. (2005). Awareness and knowledge of epilepsy among students in a Malaysian University. *Seizure*.

[16] Danesi M. A., Adetunji J. B. (1994). Use of alternative medicine by patients with epilepsy: a survey of 265 epileptic patients in a developing country. *Epilepsia*.

[17] kabir M., Iliyasu Z., Abubakar I. S., Kabir Z. S., Farinyo A. U. (2005). Knowledge, attitude and beliefs about epilepsy among adults in a Northern Nigerian urban community. *Annals of African Medicine*.

[18] Oguniyi A., Osuntokun B. O. Epidemiology of neurologic illnesses in Africa.

